# Recent developments in statistical methods for detecting genetic loci affecting phenotypic variability

**DOI:** 10.1186/1471-2156-13-63

**Published:** 2012-07-24

**Authors:** Lars Rönnegård, William Valdar

**Affiliations:** 1 Statistics Unit, Dalarna University, SE-781 70 Borlänge, Sweden; 2 Department of Animal Breeding and Genetics, Swedish University of Agricultural Sciences, SE-750 07 Uppsala, Sweden; 3 Department of Genetics and Lineberger Comprehensive Cancer Center, University of North Carolina at Chapel Hill, Chapel Hill, NC 27599-7265, U.S.A

## Abstract

A number of recent works have introduced statistical methods for detecting genetic loci that affect phenotypic variability, which we refer to as variability-controlling quantitative trait loci (vQTL). These are genetic variants whose allelic state predicts how much phenotype values will vary about their expected means. Such loci are of great potential interest in both human and non-human genetic studies, one reason being that a detected vQTL could represent a previously undetected interaction with other genes or environmental factors. The simultaneous publication of these new methods in different journals has in many cases precluded opportunity for comparison. We survey some of these methods, the respective trade-offs they imply, and the connections between them. The methods fall into three main groups: classical non-parametric, fully parametric, and semi-parametric two-stage approximations. Choosing between alternatives involves balancing the need for robustness, flexibility, and speed. For each method, we identify important assumptions and limitations, including those of practical importance, such as their scope for including covariates and random effects. We show in simulations that both parametric methods and their semi-parametric approximations can give elevated false positive rates when they ignore mean-variance relationships intrinsic to the data generation process. We conclude that choice of method depends on the trait distribution, the need to include non-genetic covariates, and the population size and structure, coupled with a critical evaluation of how these fit with the assumptions of the statistical model.

## 

Traditional approaches to mapping genes affecting quantitative traits have focused on identifying loci for which an allelic substitution shifts the phenotype of interest in a particular direction (eg, where substituting genotype AA for AG causes the phenotype to increase on average by a certain amount). Such quantitative trait loci (QTL) can be described as mean-controlling, because they are primarily observed to affect the expected, ie, average, value of individuals’ phenotypes. It is also possible, however, to detect genetic loci whose allelic substitutions are associated with an increase in variability of the phenotype about its expected value.

Figure 1a illustrates the concept, plotting phenotypes collected from a hypothetical population of 500 outbred individuals (eg, humans). When stratified by genotype at a suitable locus, the apparent effect of allelic substitution is to alter the spread of likely phenotype values: individuals carrying the AA genotype are more phenotypically similar to each other, whereas those carrying the GG genotype are more variable. To help distinguish common uses of the term “phenotypic variance”, we describe such dispersion of phenotype values around an apparent mean as “phenotypic variability”, and we denote genetic loci exhibiting the pattern in Figure 1a as “vQTL” (QTL associated with changes in phenotypic variability; [1]).

**Figure 1:**
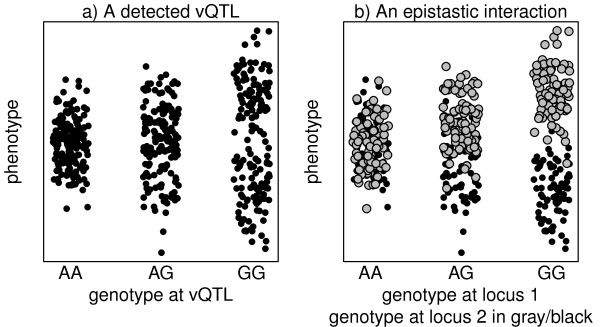
**Relation between a vQTL and an epistatic interaction.** Panel (a) plots phenotype values in arbitrary units for a population of 500 outbred individuals stratified by genotype at a hypothetical vQTL. Panel (b) shows how the pattern in (a) could have arisen through a simple (mean-controlling) epistatic interaction with a second locus, possibly on another chromosome, that segregates two genotypes (black and gray).

The biology that explains a vQTL signal can be profound, although its nature will depend on context and may require further modeling and/or experiments to characterize even broadly. Simplistically: a vQTL alerts the researcher to the presence of unmodeled statistical interactions associated with the locus. These could include interactions of potentially high order with other genes or environmental factors, and may implicate a pivotal role for the locus in maintaining phenotypic robustness and/or variability in the face of changing environment, background genetics, and temporal progression. Yet as deep as the implications of such vQTL signals may be, a crucial practical concern is how to detect them in a manner that is powerful, reliable, and robust not only to assumptions about statistical distributions but also to known features of an experiment or population that could potentially bias inference. Herein, we separate two issues: interpretation of vQTL, and statistical detection of vQTL. Both have received a recent surge of interest in the genetics literature [1-10]. We discuss the first only in brief (with references for further reading), and concentrate on the second. By providing commentary on current statistical approaches for identifying vQTL we aim to encourage continued development and thought in this area, promote the investigation of vQTL, and moderate any misalignment of statistical with experimental strategies that might lead to exotic interpretations of pedestrian results. Simply put, our focus is on methods analyzing the variance of a phenotype given a particular genotype.

### What vQTL are and where to find them

A number of recent studies [1-10] have highlighted the great potential value of identifying vQTL in humans, livestock, plants, and model organisms. One explanation for a vQTL signal is that the detected locus is involved in a gene-by-gene (ie, epistatic) interaction, as depicted in Figure 1b. Biologically, such an interaction is consistent with a variety of scenarios including the locus being a hub component in a network of genetically influenced actors, the locus being a key regulator that confers robustness of the phenotype to changes in background genetics, or a highly specific incompatibility between two QTL that destabilizes physiology. Another explanation is that the action of the locus is, under one genotype more than another, strongly affected by an unmodeled environmental exposure, eg, different weather systems associated with geography or (eg, in humans) smoking behavior, and thereby corresponds to a gene by environment interaction [3]. An explanation related to both of the above is that the locus regulates sensitivity to general changes in the environment, leading to increased variability that would manifest either between congenic individuals or within each individual at different timepoints (eg, [1,5,11]).

Although little is known about how common variance-controlling genes are in the genome or the magnitude of their contribution to trait variation in natural populations [11], clear examples of vQTL effects have been found in flies [12], humans [3], plants [10], and rats [9]. Mackay and Lyman [12] found that the coefficient of variation (CV; see later) of bristle numbers varied among chromosome substitution lines of *Drosophila melanogaster*. Paré et al. [3] found vQTL single nucleotide polymorphisms (SNPs) for levels of inflammatory biomarkers in a study of 20,000 women, which subsequent analysis showed could be explained by interactions of one locus with body mass index in the prediction of C-reactive protein (CRP) levels, and another locus with smoking behavior for levels of biomarker ICAM-1. Using publicly available GWAS data [13], Shen et al. [10] detected a vQTL for cellular control of molybdenum concentration in *Arabidopsis thaliana* located on the exon of *MOT1*. Although the level of molybdenum in *Arabidopsis* is known to be regulated by the mitochondrial molybdenum transporter encoded by the *MOT1* gene [14,15], no significant effect on the mean was found in the original GWAS [13]. Perry et al. [9] found several vQTL for urinary calcium levels in an F2 cross of genetically hypercalciuric (ie, kidney-stone forming) *Rattus norvegicus* with normocalciuric Wistar-Kyoto rats, with evidence of sex-specificity for the presence of such effects.

### Detecting vQTL is a shortcut for detecting interactions

One of the most exciting developments in this area is the use of methods that identify vQTL as a means to detect interactions. Hereby, epistasis can be detected using a fast search algorithm in one-dimensional parameter space, and G×E can be detected without the need to measure the interacting environmental effect [3,4]. We [1] have also described the close relationship between detection of vQTL and epistasis in the context of QTL studies on experimental populations. Although the scope of possible causes can be restricted through experimental design [1], the detection of a vQTL need not directly imply either the identity of the interacting factor or the nature of the detected interaction. Moreover, the absence of a vQTL effect does not imply absence of interaction effects [4]. Nonetheless, detecting vQTL can be valuable in prioritizing loci for further investigation [6], and can reduce a multi-dimensional search for epistatic QTL to a single search along the genome.

A current challenge is that statistical methods for detecting vQTL are immature relative to those for detecting mean-controlling QTL. This is in part because estimating effects on the variance is more difficult than on the mean [2], requiring greater sample sizes to obtain equivalent precision (ie, standard errors of the same width) while being more sensitive to confounding with mean effects, improperly modeled sample heterogeneity, and covariate uncertainty. The last two years have seen a flourish of papers introducing statistical techniques for detecting vQTL, and their simultaneous publication has in many cases precluded the opportunity for comparison across methods. Below we briefly survey some of these new methods, the respective trade-offs they imply, and the connections between them. We focus on methods to detect vQTL as such, and less on strategies for further characterizing interactions that might underly the detected variance heterogeneity.

### Classical non-parametric methods for detecting variance heterogeneity

Conover et al. [16] provide a detailed survey of classical methods to identify heterogeneous variance between groups. Letting each genotype class correspond to a different group provides a natural way to apply such methods to the detection of vQTL. Paré et al. [3], Struchalin et al. [4], and Deng & Paré [6] (in their GEWIST algorithm) describe procedures for detecting vQTL based on Levene’s test [16,17], presenting these as tools that help identify epistasis or G×E. Levene’s test computes a test statistic based on absolute deviations of trait values from the mean (or median [18]) within each genotype class. Significance is then judged by a simple ANOVA-like test. Fraser & Schadt [5] use the Fligner-Killeen test, which is like Levene’s test but based on ranks, to perform *in silico* mapping of vQTL for gene expression in 19 strains of mice. As non-parametric methods, both tests makes few assumptions about the sampling distribution of the data and so are robust to model misspecification [16,19].

Nonetheless, both tests (and many other such classical tests of variance heterogeneity) require that the data can be grouped into genotype classes, and they lack a natural basis for inclusion of continuous covariates. It is often necessary in GWAS and QTL studies to control statistically for non-genetic covariates such as age, body weight, and other continuous or ordered measurements, including effects that control for uneven relatedness of individuals. Indeed this can be essential to, for instance, increase power or model confounder bias. Such concerns are no less relevant in vQTL detection. The fact that methods based on Levene’s test [3,4] (or Fligner-Killeen) do not naturally model continuous covariates thus represents a limitation of their use, and in part motivates the development of the more flexible parametric and semi-parametric alternatives discussed below.

### Full parametric modeling of mean and variance

Parametric approaches are rooted in the idea that detection and interpretation is best served through specifying a generative model of the data, in this case one that describes how effects on the mean and variance combine to produce outcomes whose statistical properties approximate those of the true sampling distribution. Sorensen [20] was among the first to propose a parametric Bayesian model to detect vQTL. His Markov chain Monte Carlo (MCMC) method simultaneously fits the tested loci for mean and variance effects. A linear predictor is specified for $\log(\overset{2}{\underset{i}{\sigma }})$, where $\sigma_{i}^{2}$ is the residual variance for observation *i*. Yang et al. [8] later assessed this method in a study that included both simulations and analysis of real data on back fat thickness in pigs. More or less concurrently, we [1] developed a deterministic classical estimation procedure using Double Generalized Linear Models (DGLM [21]) that include a linear predictor for $\log(\overset{2}{\underset{i}{\sigma }})$ in a similar manner to Yang et al. [8]. DGLM estimation is fast because it merely iterates between weighted least squares. For a normally distributed trait, the DGLM iteratively fits the linear predictor for $\log(\overset{2}{\underset{i}{\sigma }})$ by taking the estimated squared residuals from the fit of a weighted linear model, using these as response variable in a second GLM (Generalized Linear Model; [22]), and then using this GLM fit to update the weights in the first stage linear model, cycling until the algorithm converges. The fitted squared residuals are also corrected for their estimation uncertainty in each iteration.

### Two-stage approximations to parametric models

Visscher & Posthuma [2] derived expressions to detect vQTL effects by working with the expectation of squared observations given non-genetic effects, and fitted these to a gamma distribution. This expectation was split into two parts: variance due to additive genetic effects on the mean, and a residual variance with a multiplicative model for the genetic effects. In terms of linear and generalized linear models, this is similar to fitting a linear model for the mean-controlling QTL and subsequently fitting a gamma GLM with log-link on the estimated squared residuals, and so is akin to a DGLM. Their method is non-iterative, however, and as such does not account for the uncertainty in estimation of residuals.

A less parametrically justifiable but more computationally convenient approach for incorporating an arbitrary set of predictors into a test for effects both in the mean and variance is to fit first a linear model for mean effects, and then fit a separate second linear model on some function of the residuals. Recently, Perry et al. [9] did this in their analysis of vQTL in a rat F2, fitting first a linear model accounting for multiple covariates and single locus effects, and then performing a separate regression on the absolute values of residuals from the initial model. Months earlier, Struchalin et al. [7] presented a method Squared residual Value Linear Modeling (SVLM), implemented in the VariABEL package (http://www.genabel.org), which fits a linear model to the squared residuals from an ordinary GWAS. These approximations trade parametric justifications for convenience and speed: when perfectly estimated, the squared residuals from the initial fit will be gamma distributed (as in the model by Visscher & Posthuma) and therefore violate the normality assumptions required by the second fit. Moreover, like the model of Visscher & Posthuma, these two methods do not account for the fact that the residuals are themselves estimated with error.

Nonetheless, we find that when the data are sizable enough to ensure that residuals are accurately estimated, SVLM and DGLM give similar performance. To illustrate this, we performed 1,000 trials of the simulation described in Struchalin et al. [7], whereby *n* = 10,000 observations are drawn from a phenotype model affected by both a SNP and an interacting factor, which in combination manifest as a vQTL. Specifically, we simulated the phenotype of individual *i*=1,…,*n* as _*yi*_∼N(_*βF*__*Fi*_ + _*βgF*_·_*gi*__*Fi*_, 1), drawing genotypes as _*gi*_∼Bin(2,0.4), and drawing the interacting factor as _*Fi*_∼N(0,1), with main effect _*βF*_=0.85 and interaction _*βgF*_=0.06 (using their notation). In this setting, where the sample is large and the genotypes are relatively well balanced, SVLM and DGLM produced almost identical p-values (correlation 0.9996) and the power at a 95% significance level was 0.82 for both methods, whereas Levene’s test produced substantially different p-values and reduced power (0.69). The false positive rates (FPR) were also assessed for the different methods for simulated standard normal trait values, _*yi*_∼N(0, 1), showing very small or no inflated FPR (Table 1).

**Table 1:** Comparison of vQTL methods

		
**Estimation method**	**Simulated distribution**	
	**Gaussian**	**Poisson**
Levene’s test	0.053	0.497
SVLM	0.063	0.389
DGLM-Gaussian	0.064	0.632
DGLM-Poisson	n.a.	0.055
		

n.a. = not applicable.

False positive rates for simulations with no interaction effects (10,000 individuals and 1,000 replicates).

### The value of simultaneously estimating mean and variance

The fully parametric models mentioned above fit both the mean and the variance simultaneously. Methods that make use of a (non-iterative) two-step approach, such as SVLM, have the disadvantage that they involve conditioning on an unknown [11]. Effects on the mean will be estimated without accounting for heteroscedasticity, which will affect the p-values for this part of the model (although in practice this may have minor consequence).

Errors in the residuals arise because these are a combination of true residuals and the under- or over-predictions of the imperfectly estimated mean part of the model. The accuracy of estimated squared residuals, that is, the correlation between true and estimated squared residuals, is given by the “hat matrix” [11,23]. Specifically, the hat matrix **H**is the matrix of linear transformation between the observed and fitted response vector (*y* and ŷ, resp.) such that ŷ=Hy. The expected value of the estimated squared residuals will be smaller than the squared true residuals and should therefore be divided by 1−_*hii*_, where _*hii*_ are the diagonal elements of **H**, referred to as “hat values” (or “leverages” [23]). We suggest that the output from simple two-step approaches should be assessed by computing the hat values for the standard GWAS model (ie, the linear model describing the SNP effect on the mean). If all hat values are small, say <0.05, there will be no need for concern since the prediction errors will have little influence on the estimates of the squared residuals.

### Extensions: dealing with genotype uncertainty

Often the genotype at a marker is not known with certainty, but is available as a probability based on surrounding marker information. This is the general case in QTL analysis (ie, linkage disequilibrium mapping), and is typical in GWAS where genotypes are imputed. In ordinary QTL analysis and GWAS, genotype probabilities can be used in place of the observed genotypes (possibly after reformulation) as predictors in a linear model of the phenotype [24-26]. In such cases, the residual variance will typically be inflated because uncertainty about the genotype will manifest as additional error in the residual part of the model [27,28]. In regression-based methods for vQTL detection, including DGLM and SVLM, genotype probabilities can similarly be incorporated as predictors, which is not possible in Levene’s test. An application highlighted in [7] is imputation of genotype values in GWAS. There is an obvious risk that the inflated residual variance caused by genotype uncertainty might inflate the false discovery rate for vQTL because the estimated squared residuals are used as response variable. We evaluated this risk for F2 and Collaborative Cross designs in QTL analysis (see Figure 2 and Supplementary Theory part 3 in [1]) but found no apparent inflation in false positives. For GWAS using either DGLM or SVLM, however, no such thorough assessment has been performed.

### Extensions: incorporating population structure and polygenic effects

A common problem in GWAS is accounting for effects of population structure. We [1] have proposed an approach where the polygenic random effects are fitted simultaneously with the rest of the model using hierarchical generalized linear models (HGLM, [29,30]), implemented in the R package hglm available on CRAN [31]. HGLM is a direct extension of DGLM that includes random effects in the mean part of the model and can be extended to include random effects influencing the residual variance [11,32]. Struchalin et al. [7] propose an alternative two-step approach, using SVLM after pre-correction for polygenic random effects, with this pre-correction performed in a similar way to that applied in their GRAMMAR approach [33] whereby residuals from polygenic model are used as the response for subsequent analysis. As with SVLM, this extension comes at the expense of conditioning on further unknowns (see also Discussion in [34]). Specifically, it involves subtracting from the phenotype point estimates of the polygenic effects, which themselves are known with error. Such shortcuts can be extremely valuable because they allow faster, more tractable analyses. But when using them it is also important to consider under which circumstances their accumulated effects might bias results, by, for instance, checking the values in the hat matrix.

### Model misspecification, the scale of measurement, and the coefficient of variation

One of the greatest challenges in the vQTL detection is how to choose the scale of measurement on which to draw conclusions about estimated effects. When we detect a SNP whose alleles increase both the mean and the variance, should we interpret it as a vQTL with a significant marginal effect, or a mean-controlling QTL for a trait that was analyzed on the wrong scale? Suppose, for instance, that we had a cylindrical organism (such as a snake or a worm) whose body width increases with its body length, and we have a gene with a strong additive effect on body length, which is itself normally distributed. A GWAS on the volume of this cylindrical organism is likely to detect a QTL controlling both mean and the variance, despite the fact that a simpler explanation is available.

To circumvent such ambiguities, some researchers have used the coefficient of variation (CV; the standard deviation divided by the mean) to detect genotypic effects on the variance [12]. This approach is highly applicable for a trait that is positive-valued, and for which (under a null model) the standard deviation would be expected to increase with the mean. A more flexible remedy is to find a transformation of the trait for which the residuals under a mean-effects model are approximately Gaussian (and thereby symmetric), and estimate genotypic effects on this transformed phenotype. This helps guard against vQTL interpretations that could be otherwise explained by a combination of mean effects and transformation. We [1] and Yang et al. [35] promote the use of the Box-Cox procedure to help guide such transformations, with Yang et al. making this procedure an intrinsic and simultaneously estimated component of their Bayesian model. The QTL and vQTL effects detected on a transformed scale can be back-transformed to the original scale to study their effects on the scale of interest.

In some cases, the trait is known to be well approximated by a distribution that has a known mean-variance relationship, such as the Poisson or gamma distribution. In such cases, it will often be preferable to model that distribution explicitly and define vQTL parameters as those that alter the variance in a way not already anticipated by concomitant effects on the mean. DGLMs provide a natural basis for such models when the known sampling distribution is member of the exponential family [1].

To illustrate the effect of a misspecified distribution on vQTL detection, we consider the following simulation study in which vQTL-detection methods are applied to a non-normal phenotype. Let the phenotype _*Yi*_ of individual *i* be distributed as _*Yi*_∼Poisson(_*μi*_), where the individual’s genotype _*gi*_ affects the phenotype through the relation $E(Y_{i})=\mu_{i}=\exp(\mu +\beta_{g}g_{i})$, with *μ*=−1,_*βg*_=0.05. This set up is similar to that used in Struchalin et al. [7] with the difference that whereas they use a normally distributed phenotype subject to interactions (ie, a true vQTL signal), we use a Poisson distribution with no interaction effect (ie, no vQTL signal). We performed 1000 simulation trials. In each, we generated 10,000 phenotypes and tested the significance of a vQTL effect using three methods: SVLM, DGLM with a Gaussian response (DGLM-normal), and DGLM with a Poisson response (DGLM-Poisson). At a 95% significant level, Levene’s test, SVLM and DGLM-normal had strongly inflated FPR, whereas DGLM-Poisson had a more reasonable FPR of 5.5% (Table 1). Hence, SVLM or a misspecified DGLM are likely to produce a large proportion of false positives for a trait whose variance is a function of its mean. Further research is required to distinguish trait values generated by a distribution where the variance explicitly depends on the mean from a process where the variance is controlled by a vQTL.

### Connections to related literature: relationship QTL, and breeding livestock for uniformity

One interesting possible cause of a vQTL signal arises when a QTL affecting a primary trait of interest also affects another (secondary) trait in a way that can restrict variation of the primary trait (eg, in closely related morphological phenotypes). Cheverud et al. [36] develop the idea of a QTL affecting multiple traits in this and other ways, referring to such QTL as relationship QTL (relQTL; see also [37,38]). Their approach for detecting relQTL requires that the interacting traits are known a priori. The concepts of relQTL and vQTL overlap to a degree in that a relQTL could manifest as a vQTL. A fundamental conceptual difference between the two is that vQTL may also arise through other means, and a fundamental practical difference is that vQTL detection does not require prior knowledge of the interacting factor.

We further note that several semi-parametric and fully parameterized estimation methods have been developed for animal breeding purposes over the last couple of decades. These methods incorporate the additive relationships between individuals to assess the possibility of reducing phenotypic variability in breeding programs. They are not directly applicable in GWAS nor QTL analysis but the modeling approach is very similar to that of vQTL detection. A future possibility is to perform standard GWAS using estimated breeding values for variability as response. A deeper understanding of new methods for vQTL detection could therefore be obtained by relating these to the already existing literature on genetic heterogenity in animal breeding (see [11] and refs therein).

### Conclusion

Studies that develop statistical methods for vQTL detection, as well as ones that exemplify their use, feed a growing interest in a fascinating emerging area of complex trait genetics. We have reviewed some of the new methods for detecting vQTL, and provided commentary on their respective trade-offs. Classical group-based non-parametric methods such as Levene’s test can be robust to model misspecification but lack flexibility and the scope to include continuous covariates, genotype probabilities (eg imputed genotypes) or random polygenic effects. Parametric methods fully account for the uncertainty of fitted parameters in both the mean and the variance parts of the model, and also allow fitting of covariates and random polygenic effects in both parts, but are more sensitive to modeling assumptions. Semi-parametric or two-stage approaches can be faster but come at the price of shortcuts that in some cases can lead to bias. The choice of method depends on the trait distribution, the need to include non-genetic covariates, and the population size and structure. We advise that the assumptions of the chosen model be evaluated and compared with those of alternatives, and we expect that if this is performed in a careful manner then these methods could be of great use in both the analysis of GWAS and QTL mapping data.

## Author’s contributions

Both authors wrote the text and approved the final version of the manuscript.
